# Trends in the rate of regular exercise among adults: results from chronic disease and risk factor surveillance from 2010 to 2018 in Jiangsu, China

**DOI:** 10.3389/fpubh.2023.1089587

**Published:** 2023-06-15

**Authors:** Jian Su, Jinxin Yu, Yu Qin, Ran Tao, Jie Yang, Shurong Lu, Jinyi Zhou, Ming Wu

**Affiliations:** ^1^Department of Non-communicable Chronic Disease Control, Provincial Center for Disease Control and Prevention, Nanjing, Jiangsu, China; ^2^Department of Epidemiology, Center for Global Health, School of Public Health, Nanjing Medical University, Nanjing, China

**Keywords:** exercise, physical activity, surveillance, trends, China

## Abstract

**Objective:**

The aims of this study were to estimate the rates of regular exercise and its trends among the adult population in Jiangsu, from 2010 to 2018, China, and to assess associations with sociodemographic factors.

**Methods:**

Chronic disease and risk factor surveillance data from adults aged ≥18 years were gathered in Jiangsu Province from 2010 to 2018. Rates of regular exercise were calculated after post-stratification weighting, and time trends were compared among participants with different characteristics, including gender, age, urban–rural region, educational level, occupation, annual household income, body mass index (BMI), baseline self-reported chronic diseases, smoking status, alcohol consumption, and region. Multivariable logistic regression analyses were performed to assess the associations of sociodemographic characteristics with regular exercise.

**Results:**

A total of 33,448 participants aged 54.05 ± 14.62 years and 55.4% female (8,374 in 2010, 8,302 in 2013, 8,372 in 2015, and 8,400 in 2018) were included in this study. The weighted rate of regular exercise was 12.28% (95% confidence interval [CI]: 9.11–15.45%) in 2010 and 21.47% (95% CI, 17.26–25.69%) in 2018, showing an overall increasing trend (*P* for trend = 0.009). Nevertheless, stratification analysis showed that the regular exercise rate decreased from 33.79% in 2010 to 29.78% in 2018 among retired adults. Significant associations were observed between regular exercise and age >45 years (45– < 60 years, odds ratio [OR]: 1.24, 95% CI: 1.14–1.34; ≥60 years, OR: 1.20, 95% CI: 1.08–1.34), urban residence (OR: 1.43, 95% CI: 1.32–1.54), higher education (primary, OR: 1.30, 95% CI: 1.16–1.46; secondary, OR: 2.00, 95% CI: 1.79–2.25; college or higher, OR: 3.21, 95% CI: 2.77–3.72), occupation (manual work, OR: 1.52, 95% CI: 1.33–1.73; non-manual work, OR: 1.69, 95% CI: 1.54–1.85; not working, OR: 1.22, 95% CI: 1.03–1.44; retired, OR: 2.94, 95% CI: 2.61–3.30), higher income (¥30,000– < ¥60,000, OR: 1.16, 95% CI: 1.06–1.28; ≥¥60,000, OR: 1.20, 95% CI: 1.10–1.32), higher BMI (overweight, OR: 1.12, 95% CI: 1.05–1.20), self-reported chronic disease at baseline (OR: 1.24, 95% CI:1.16–1.33), former smoking (OR: 1.15, 95% CI: 1.01–1.31) and ever (30 days ago) drinking (OR: 1.20, 95% CI: 1.11–1.29).

**Conclusion:**

The rate of regular exercise among adults in Jiangsu Province was low, but this rate increased by 9.17% from 2010 to 2018, showing an upward trend. There were differences in the rate of regular exercise among different sociodemographic factors.

## Introduction

Physical inactivity is an established risk factor for non-communicable diseases such as hypertension, diabetes, cardiovascular diseases, cancer, and chronic obstructive pulmonary disease (COPD) (1–4). Eliminating physical inactivity would enhance global population life expectancy by 0.68 years (5). Physical inactivity also leads to higher health care costs (6). Despite such negative effects of physical inactivity, a large proportion of Chinese adults do not engage in leisure-time physical activity. According to the findings of the China Health and Nutrition Survey, the age-standardized leisure-time physical activity rate of Chinese adults increased from 7.13% in 2000 to 11.79% in 2011, but then dropped to 7.33% in 2015 (7).

Other studies have found that leisure-time physical activity is associated with reduced risk of all-cause, cardiovascular, type 2 diabetes, and cancer mortality (8, 9), and regular exercise is essential for both physical and mental well-being (10). The World Health Organization 2020 guidelines on physical activity and sedentary behavior recommended regular aerobic exercise for all age groups (11). The most recent national fitness plan (2021–2025), which was announced in August 2021, highlighted the benefits of regular leisure-time physical activity in promoting population health and quality of life (12).

Existing Chinese studies mostly described regular exercise rates in a single year, rather than analyzing changes in trends in regular exercise rates (13, 14). Previous studies have assessed trends in regular exercise rates in Western countries, but definitions of regular exercise rates are not uniform across countries (15, 16). The population in China differs from that in developed countries in terms of the importance of healthy lifestyles, the availability of infrastructure facilities, and the hosting of sports events, but the trends in the rate of regular exercise in the Chinese population may be useful to other developing countries in implementing physical activity programs. Chinese population is changing from a developing country to a developed country. The shift in physical activity is large. For example, the level of leisure-time physical activity showed an increasing trend and the consumption of physical exercise increased (17). As such, the study of trends in physical activity in Chinese population has implications for other developing countries. In addition, “The Health China 2030 Plan Outline” (18) and “Medium- and long-term planning for the prevention and treatment of chronic diseases in China (2017–2025)” (19) propose to increase the proportion of regular physical activity to reduce the burden of chronic diseases. Regular exercise rate and socioeconomic status interact with each other (20). Individuals with higher socioeconomic status are more likely to be exercise, and individuals with lower socioeconomic status are more often engaged in heavy workloads, longer work hours, and more night work, with less time and energy for physical activity (21). Regular exercise leads to more efficient learning, resulting in higher education level, higher income, and higher socioeconomic status (22). In addition, few previous studies have examined the association between regular exercise rates and socioeconomic factors. Investigating the association between regular exercise and socioeconomic status can help develop physical activity promotion interventions that target specific socioeconomic status groups.

## Methods

### Survey design and participants

Chronic disease and risk factor surveillance (CDRFS) data were collected in Jiangsu Province. The CDRFS is a series of provincially representative cross-sectional surveys on chronic diseases and behavioral risk factors in the general population. In 2010, 2013, 2015, and 2018, the Department of Non-communicable Chronic Disease Control, Jiangsu Provincial Center for Disease Control and Prevention, carried out four surveillance programs on chronic diseases and their risk factors among permanent residents in Jiangsu Province. The CDRFS selected 14 disease surveillance points in Jiangsu Province, covering 13 prefecture-level cities. The subjects of the CDRFS were the general population from the community who had been living in their current residence for at least 6 months and were at least 18 years old. A multistage stratified cluster sampling method was used for the CDRFS. In the first stage, townships (rural) or subdistricts (urban) were randomly selected with Probability Proportionate to Size (PPS) sampling. In the surveys of 2010 and 2013, four townships or subdistricts were selected. In the 2015 and 2018 surveys, three townships or subdistricts were selected. In the second stage, villages (rural) or residential areas (urban) were randomly selected with PPS sampling. Three villages or residential areas were selected in 2010 and 2013, and two were selected in 2015 and 2018. In the third stage, each selected village or residential area was divided into groups of about 50 households, based on existing villager/resident groups in the village or residential area. One group was selected with simple random sampling. In the fourth stage, within each selected household, in the 2010 and 2013 surveys, the Kish method was used to select one permanent resident aged 18 years or older. In the surveys of 2015 and 2018, all permanent residents aged 18 years or older in the household were invited. The CDRFS system has been proven to be provincially representative and can adequately describe the epidemiological characteristics of population health indicators in Jiangsu Province (23, 24). Details of the design, objectives, and survey methods of the CDRFS have been described previously (25, 26).

Each round of the 2010–2018 China Chronic Diseases and Risk Factors Surveillance Survey used independent sampling, with different participants for each survey. From 2010 to 2018, four surveys were conducted. A total of 34,065 community residents aged 18 years and older were surveyed, including 8,400 in 2010, 8,399 in 2013, 8,689 in 2015, and 8,577 in 2018. All participants provided informed consent.

### Data collection

Data were collected through questionnaire interviews, anthropometric measurements, and laboratory tests. In each survey, questionnaire interviews included information on sociodemographic (sex, age, residence, region, education, occupation, annual household income, and baseline self-reported chronic diseases) and lifestyle risk factors (physical activity, smoking status, and alcohol consumption). Anthropometric measurements included standing height and weight. Body mass index (BMI) was calculated as weight in kilograms divided by height in meters squared. All were collected by trained and qualified personnel. The questionnaire was formulated and revised by the Chronic Disease Center of the Chinese Center for Disease Control and Prevention. All questionnaire data were required to be entered twice. The Jiangsu Provincial Center for Disease Control and Prevention conducted on-site supervision of surveillance points, randomly checked and reviewed the questionnaires, and audited the final reported questionnaires and database.

Information on physical activity was self-reported by participants using the Global Physical Activity Questionnaire (GPAQ), which was developed by the World Health Organization for physical activity surveillance in countries and has good reliability and validity (27). GPAQ covered three components (intensity, duration, and frequency) and three domains (work-related, transportation-related, and leisure-time physical activity). For leisure-time physical activity, participants were asked “Do you engage in vigorous-intensity activity that lasts at least 10 min and causes a significant increase in breathing and heart rate?.” If they answered yes, they were then asked how many days in a typical week and how many minutes per day they performed the activity. The same questions were asked for moderate-intensity activity. Regular exercise was defined as engaging in vigorous and/or moderate leisure-time physical activity at least 3 times a week for at least 10 min per session (28, 29).

Occupations were classified into agriculture-related work (agriculture, forestry, animal husbandry, and fishery), manual work (production and transportation equipment operators and related personnel), non-manual work (business service personnel, persons in charge of institutions, officials and related personnel, professional and technical personnel, soldiers, other workers, students, and houseworkers), not working, and retired. BMI was classified into underweight (<18.5 kg/m^2^), normal (18.5 kg/m^2^–23.9 kg/m^2^), overweight (24.0 kg/m^2^–27.9 kg/m^2^), and obese (≥28.0 kg/m^2^) (30). Self-reported chronic diseases included hypertension, diabetes, dyslipidemia, myocardial infarction, stroke, COPD, and cancer. Self-reported chronic disease at baseline was defined as having at least one chronic disease.

### Statistical analysis

Participants with missing data on physical activity (*n* = 301) and missing data on either body weight or height (*n* = 316) across all four surveys were excluded, leaving 33,448 participants included in this analysis. Post-stratification weighting was used to weight the data according to the 2010 Jiangsu provincial census data, to allow comparison of data across surveys from 2010 to 2018. The rate of regular exercise was analyzed by gender (female vs. male), age (18– < 45, 45– < 60, ≥60 years), residence (rural vs. urban), education (no formal education, primary, secondary, college or higher), occupation (agriculture-related, manual work, non-manual work, not working, retired), annual household income (<¥30,000, ¥30,000– < ¥60,000, ≥¥60,000, refusing to answer/unknown), BMI (underweight, normal, overweight, obesity), and self-reported chronic disease at baseline (no vs. yes). The proc. surveyfreq procedure was used to estimate the rate of regular exercise, with standard error and 95% confidence interval (CI) values.

Trends in the rate of regular exercise over time were estimated using a univariate logistic regression model, which included the year of each survey as a continuous variable. A multivariable logistic regression model was used to examine associations between sociodemographic factors and regular exercise. To reduce the influence of differences in sociodemographic factors across years on the associations between regular exercise and sociodemographic factors, adjustment was made for the year of each survey in the multivariable logistic regression model combining the four surveys. A two-tailed *p* value <0.05 was considered statistically significant. All analyses were conducted using SAS version 9.4.

## Results

A total of 33,448 individuals (mean age, 54.05 years [standard deviation, 14.62 years]; 18,544 [55.4%] female) were included in this analysis. A significant difference was found in population distribution across the four survey rounds. Participants were primarily urban residents (59.0%) and residents without chronic disease at baseline (64.2%). A total of 46.3% of the sample had a secondary school degree and 45.8% undertook non-manual work. The percentages of participants with higher household income (≥￥60,000) and obesity increased between 2010 and 2018 (Table 1).

**Table 1:** Characteristics of participants, CDRFS 2010–2018.^a^

Characteristic	No. (%) of participants					*P* value
	2010–2018 (*n* = 33,448)	2010 (*n* = 8,374)	2013 (*n* = 8,302)	2015 (*n* = 8,372)	2018 (*n* = 8,400)	
Gender						
Female	18,544 (55.4)	4,586 (54.8)	4,737 (57.1)	4,542 (54.3)	4,679 (55.7)	0.002
Male	14,904 (44.6)	3,788 (45.2)	3,565 (42.9)	3,830 (45.7)	3,721 (44.3)	
Age, years						
18- < 45	8,815 (26.4)	2,751 (32.9)	2,460 (29.6)	2,024 (24.2)	1,580 (18.8)	<0.001
45- < 60	12,178 (36.4)	3,046 (36.4)	3,244 (39.1)	2,856 (34.1)	3,032 (36.1)	
≥60	12,455 (37.2)	2,577 (30.8)	2,598 (31.3)	3,492 (41.7)	3,788 (45.1)	
Residence						
Rural	13,727 (41.0)	3,591 (42.9)	2,939 (35.4)	3,580 (42.8)	3,617 (43.1)	<0.001
Urban	19,721 (59.0)	4,783 (57.1)	5,363 (64.6)	4,792 (57.2)	4,783 (56.9)	
Education						
No formal education	6,435 (19.2)	1,732 (20.7)	1,596 (19.2)	1,541 (18.4)	1,566 (18.6)	<0.001
Primary	8,653 (25.9)	2,278 (27.2)	2,138 (25.8)	2,086 (24.9)	2,151 (25.6)	
Secondary	15,481 (46.3)	3,845 (45.9)	3,966 (47.8)	3,785 (45.2)	3,885 (46.2)	
College or higher	2,879 (8.6)	519 (6.2)	602 (7.3)	960 (11.5)	798 (9.5)	
Occupation						
Agriculture-related	9,047 (27.0)	2,699 (32.2)	2,875 (34.6)	1,863 (22.3)	1,610 (19.2)	<0.001
Manual work	2,978 (8.9)	882 (10.5)	945 (11.4)	610 (7.3)	541 (6.4)	
Non-manual work	15,313 (45.8)	3,725 (44.5)	3,416 (41.1)	3,986 (47.6)	4,186 (49.8)	
Not working	1,783 (5.3)	207 (2.5)	321 (3.9)	541 (6.5)	714 (8.5)	
Retired	4,327 (12.9)	861 (10.3)	745 (9.0)	1,372 (16.4)	1,349 (16.1)	
Annual household income, ¥						
<30,000	8,463 (25.3)	3,735 (44.6)	1,524 (18.4)	1,704 (20.4)	1,500 (17.9)	<0.001
30,000- < 60,000	9,019 (27.0)	2,737 (32.7)	2,624 (31.6)	1,950 (23.3)	1,708 (20.3)	
≥60,000	11,426 (34.2)	1,098 (13.1)	2,780 (33.5)	3,571 (42.7)	3,977 (47.3)	
Refuse to answer/Unknown	4,540 (13.6)	804 (9.60)	1,374 (16.6)	1,147 (13.7)	1,215 (14.5)	
BMI categories^b^						
Underweight	711 (2.1)	208 (2.5)	194 (2.3)	155 (1.9)	154 (1.8)	<0.001
Normal	14,038 (42.0)	3,866 (46.2)	3,592 (43.3)	3,297 (39.4)	3,283 (39.1)	
Overweight	13,264 (39.7)	3,159 (37.7)	3,246 (39.1)	3,445 (41.1)	3,414 (40.6)	
Obesity	5,435 (16.2)	1,141 (13.6)	1,270 (15.3)	1,475 (17.6)	1,549 (18.4)	
Self-reported chronic disease						
No	21,467 (64.2)	6,025 (71.9)	5,702 (68.7)	5,133 (61.3)	4,607 (54.8)	<0.001
Yes	11,981 (35.8)	2,349 (28.1)	2,600 (31.3)	3,239 (38.7)	3,793 (45.2)	
Smoking						
Never	23,007 (68.8)	5,580 (66.6)	5,872 (70.7)	5,702 (68.1)	5,853 (69.7)	<0.001
Former	2,160 (6.46)	523 (6.25)	411 (4.95)	625 (7.47)	601 (7.15)	
Current	8,281 (24.8)	2,271 (27.1)	2,019 (24.3)	2,045 (24.4)	1,946 (23.2)	
Drinking						
Never	21,161 (63.3)	5,484 (65.5)	5,626 (67.8)	4,992 (59.6)	5,059 (60.2)	<0.001
Ever, 30 days ago	9,463 (28.3)	2,293 (27.4)	2,148 (25.9)	2,497 (29.8)	2,525 (30.1)	
Ever, within 30 days	2,819 (8.43)	597 (7.13)	528 (6.36)	881 (10.5)	813 (9.68)	
Region						
South	14,354 (42.9)	3,592 (42.9)	3,580 (43.1)	3,617 (43.2)	3,565 (42.4)	0.956
Central	7,176 (21.5)	1,798 (21.5)	1,776 (21.4)	1,800 (21.5)	1,802 (21.5)	
North	11,918 (35.6)	2,984 (35.6)	2,946 (35.5)	2,955 (35.3)	3,033 (36.1)	

BMI, body mass index; CDRFS, chronic disease and risk factor surveillance.

a Data are expressed as unweighted number of participants and unweighted percentages.

b Underweight (<18.5 kg/m^2^), normal (18.5 kg/m^2^–23.9 kg/m^2^), overweight (24.0 kg/m^2^–27.9 kg/m^2^), and obesity (≥28.0 kg/m^2^).

**Figure 1:**
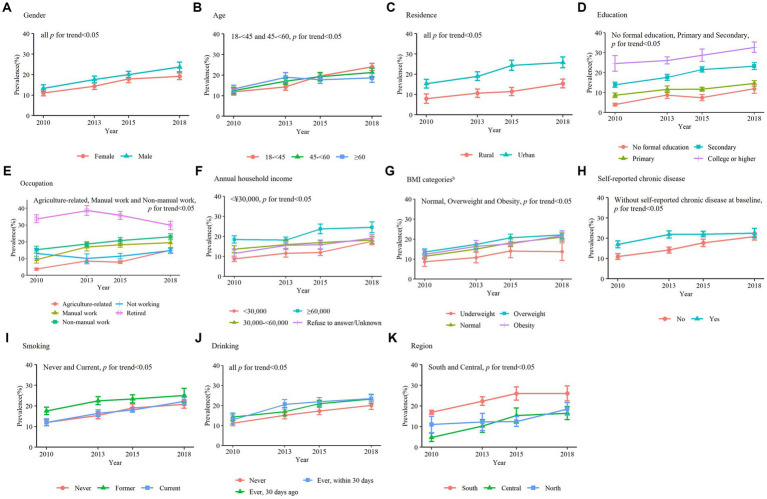
Trends in weighted rate of regular exercise, CDRFS 2010-2018^a^. BMI, body mass index; CDRFS, chronic disease and risk factor surveillance. ^a^Data were weighted to be provincially representative in each category and error bars indicate 95% CIs. ^b^Underweight (<18.5 kg/m^2^), normal (18.5 kg/m^2^-23.9 kg/m^2^), overweight (24.0 kg/m^2^-27.9 kg/m^2^), and obesity (≥28.0 kg/m^2^).

From 2010 to 2018, the overall weighted rate of regular exercise increased by 9.17% from 12.28% (95% CI: 9.11–15.45%) to 21.47% (95% CI: 17.26–25.69%) (*P* for trend = 0.009). Trends in the overall group and for each gender, residence, and baseline self-reported chronic disease groups remained similar. Among the different age groups, the rate of regular exercise was highest among adults aged ≥60 years in 2010, while the rate among adults aged ≥60 years in 2018 was the lowest. The rate of regular exercise increased with higher education and household income. Regular exercise rates were highest among overweight adults and lowest among underweight adults. The rate of regular exercise among retirees decreased from 33.79% (95% CI: 28.61–38.97%) in 2010 to 29.78% (95% CI: 24.21–35.36%) in 2018. In each year, however, retirees still had the highest rate of regular exercise among all occupations (Figure 1; Supplementary Table S1).

Various sociodemographic factors were positively associated with the rate of regular exercise. After multivariable adjustment, a significantly higher estimated regular exercise rate was observed among participants aged 45– < 60 years, those residing in urban areas, individuals with a college degree or higher, participants who were retired, individuals with annual household income ≥￥60,000, overweight participants, those with self-reported chronic diseases at baseline, ever drinkers, and residents of south Jiangsu Province. Compared with participants in the 18– < 45 years old age group, those who were 45– < 60 years old had a 24% higher regular exercise rate (OR: 1.24, 95% CI: 1.14–1.34). Compared with rural residents, the OR for the regular exercise rate for urban residents was 1.43 (95% CI: 1.32–1.54). The rate of regular exercise was higher in participants who were retired than in participants with agriculture-related jobs (OR: 2.94, 95% CI: 2.61–3.30). Relative to participants with a household income <¥30,000, the OR for those in the ¥30,000– < ¥60,000 was 1.20 (95% CI: 1.10–1.32). Further, participants with higher education and higher BMI tended to exercise more regularly (*P* for trend <0.001). Self-reported chronic disease at baseline was associated with a 24% higher regular exercise rate (OR: 1.24, 95% CI: 1.16–1.33) (Table 2). The rate of regular exercise was 20% higher among those who had consumed alcohol in the previous 30 days relative to those who had never consumed alcohol (OR = 1.20, 95% CI: 1.11–1.29). The association between sociodemographic factors and regular exercise according to each survey was similar to that of the overall survey (Supplementary Table S2). We observed that rural participants with college degrees or higher had higher rates of regular exercise than rural participants without formal education (OR = 4.90, 95% CI: 3.79–6.30; *P*_interaction_ = 0.001). We did not observe an interaction between smoking and BMI (Supplementary Figures S1, S2).

**Table 2:** Association between regular exercise (yes vs. no) and sociodemographic factors using pooled data of CDRFS 2010–2018^a^ (OR and 95% CI).

Characteristic	Counts	Rate of regular exercise (%, 95 CI)	OR (95% CI)	*P* value
Gender				
Female	2944/18544	15.46 (12.56–18.37)	ref	
Male	2736/14904	18.46 (15.57–21.36)	0.96 (0.88–1.05)	0.414
Age, years				
18- < 45	1428/8815	16.46 (13.45–19.47)	ref	
45- < 60	2105/12178	17.45 (14.47–20.44)	1.24 (1.14–1.34)	<0.001
≥60	2147/12455	17.36 (14.52–20.20)	1.20 (1.08–1.34)	<0.001
*P* for trend^b^			<0.001	
Residence				
Rural	1462/13727	11.24 (7.47–15.02)	ref	
Urban	4218/19721	20.83 (16.97–24.69)	1.43 (1.32–1.54)	<0.001
Education				
No formal education	542/6435	7.82 (5.23–10.40)	ref	
Primary	1077/8653	11.47 (9.31–13.63)	1.30 (1.16–1.46)	<0.001
Secondary	3190/15481	18.78 (16.35–21.20)	2.00 (1.79–2.25)	<0.001
College or higher	871/2879	28.58 (24.54–32.61)	3.21 (2.77–3.72)	<0.001
*P* for trend^c^			<0.001	
Occupation				
Agriculture-related	730/9047	7.85 (5.85–9.85)	ref	
Manual work	472/2978	15.16 (12.43–17.88)	1.52 (1.33–1.73)	<0.001
Non-manual work	2793/15313	19.44 (16.89–21.99)	1.69 (1.54–1.85)	<0.001
Not working	220/1783	12.54 (9.95–15.14)	1.22 (1.03–1.44)	0.017
Retired	1465/4327	34.04 (30.90–37.19)	2.94 (2.61–3.30)	<0.001
Annual household income, ¥				
<30,000	938/8463	11.25 (8.49–14.01)	ref	
30,000- < 60,000	1500/9019	15.68 (12.90–18.47)	1.16 (1.06–1.28)	0.001
≥60,000	2539/11426	21.99 (18.39–25.60)	1.20 (1.10–1.32)	<0.001
Refuse to answer/Unknown	703/4540	15.82 (12.87–18.77)	1.04 (0.93–1.17)	0.449
*P* for trend^d^			0.170	
BMI categories^e^				
Underweight	83/711	11.52 (8.73–14.30)	0.69 (0.54–0.87)	0.003
Normal	2244/14038	15.91 (13.16–18.65)	ref	
Overweight	2425/13264	18.34 (15.21–21.48)	1.12 (1.05–1.20)	0.001
Obesity	928/5435	17.37 (13.87–20.86)	1.05 (0.96–1.15)	0.250
*P* for trend^f^			0.010	
Self-reported chronic disease				
No	3224/21467	15.36 (12.61–18.10)	ref	
Yes	2456/11981	21.08 (18.12–24.05)	1.24 (1.16–1.33)	<0.001
Smoking				
Never	3835/23007	16.61 (13.62–19.61)	ref	
Former	463/2160	22.21 (18.98–25.43)	1.15 (1.01–1.31)	0.039
Current	1382/8281	16.81 (14.21–19.41)	0.88 (0.81–0.97)	0.011
Drinking				
Never	3367/21161	15.56 (12.67–18.45)	ref	
Ever, 30 days ago	1755/9463	18.80 (15.59–22.02)	1.20 (1.11–1.29)	<0.001
Ever, within 30 days	558/2819	20.04 (17.03–23.04)	1.16 (1.04–1.29)	0.008
Region				
South	3317/14354	22.66 (18.22–27.10)	ref	
Central	791/7176	11.40 (6.64–16.17)	0.62 (0.57–0.68)	<0.001
North	1572/11918	13.39 (6.71–20.07)	0.94 (0.87–1.02)	0.143

BMI, body mass index; CDRFS, chronic disease and risk factor surveillance.

a Unweighted estimates and 95%CIs were estimated for the overall survey.

b *P* for trend over age was calculated using the median value of each category as a continuous variable.

c *P* for trend over education was calculated using education level as a continuous variable.

d *P* for trend over annual household income was calculated using the median value of each category as a continuous variable.

e Underweight (<18.5 kg/m^2^), normal (18.5 kg/m^2^–23.9 kg/m^2^), overweight (24.0 kg/m^2^–27.9 kg/m^2^), and obesity (≥28.0 kg/m^2^).

f *P* for trend over BMI categories was calculated using the median value of each category as a continuous variable.

## Discussion

Using provincially representative data from the CDRFS in Jiangsu, China, from 2010 to 2018, we analyzed the weighted rate of regular exercise, estimated its trend over time, and examined associations of regular exercise with demographic factors. Our analysis showed that the regular exercise rate of adults in Jiangsu Province was 16.02% in 2013, which was higher than the national average level (15.0%) (29). Provinces with similar GDP to Jiangsu Province, e.g., Shandong Province (19.8%) (14), had a higher rate of regular exercise than Jiangsu Province. Provinces with lower GDP than Jiangsu Province, e.g., Gansu Province (13.95%) (13), had a lower rate of regular exercise than Jiangsu Province. Therefore, we speculate that the economy is one of the factors affecting the regular exercise rate. This part needs to be further explored. These findings demonstrate that health policies relevant to Jiangsu Province need to be developed and targeted measures implemented to increase the rate of regular exercise. Additionally, our research demonstrates that the regular exercise rate in Jiangsu Province increased by 9.17% from 2010 to 2018. A study in Beijing found that the regular exercise rate in the city increased by 8.14% from 2008 to 2017 (31, 32). Nevertheless, data reported from Iran and Madrid indicated that the trends in leisure-time physical activity declined, possibly due to increased sedentary time and obesity rates (33, 34).

The rate of regular exercise among retirees generally showed a downward trend over time; however, the rate of regular exercise among retirees was still relatively high compared to that of participants in the other occupation groups, and retirement was positively associated with regular exercise. The decline in the regular exercise rate among retirees could be attributed to the fact that China is transforming into an aging nation and the older adult population is growing rapidly (35). The aging of society makes it more likely that retirees continue working, resulting in a lack of time for leisure-time physical activity (36). Additionally, the increase in older adults taking care of their grandchildren in China has led to a decrease in the time they spend on exercise (37). The average retirement age is 60. Compared with non-retirees under 45 years old, retired people have less stress from study, work, and life and take more exercise (38). We observed a much higher rate of regular exercise in urban than in rural areas, as well as significant increases in regular exercise rates in both areas, consistent with a previous study (39). The disparity between areas may primarily be attributable to the fact that rural areas are less likely to have facilities associated with leisure-time physical activity, such as parks and green spaces (40). Additionally, residents in urban areas have higher incomes and are increasingly concerned about quality of life, and thus exercise more frequently (41).

Positive associations of regular exercise with educational and income levels were observed in our study, consistent with other studies showing that education and income are positive influences on leisure-time physical activity (20, 42). Some previous studies (43, 44) have reported an inverse relationship between BMI and leisure-time physical activity, which contradicts our findings. In our study, overweight adults had the highest regular exercise rate. The possible reasons for these different findings might be that Jiangsu Province implemented “The Outline of the National Fitness Program 2001–2010” to improve fitness publicity for people with overweight and obesity (45). The number of people who consciously participate in exercise increased, with running, square dancing, climbing mountains, and fitness becoming fashionable. Further, individuals with overweight and obesity have greater motivation to take part in physical activity with the aim of weight management and improving their appearance (46, 47). In addition, being overweight may not mean obesity, but may be an increase in muscle mass from exercise (48). Exercise increases muscle mass, and muscle is denser than fat, so people with more muscle mass at the same weight may have a higher BMI (49). Follow-up studies should focus on indicators of both waist circumference and visceral fat.

Multivariable logistic regression analyses suggested that lack of regular exercise was more severe among individuals who were younger, lived in a rural area, had a lower education level, participated in agriculture-related work, and had a lower income. In summary, people in socially/economically disadvantaged positions were more likely to have a low regular exercise rate. In a recent Swedish study, economic stress was associated with low leisure-time physical activity and the strength of the association increased sharply with higher levels of economic stress (50). Therefore, targeted interventions for the abovementioned groups should be created, to encourage them to take more exercise (11).

This study provides data support for the government to develop physical activity policies. The government can launch educational campaigns and awareness programs to promote the benefits of regular exercise and encourage citizens to adopt a more active lifestyle. The government can also improve infrastructure and recreational exercise facilities to encourage citizens to be physically active. Society can support community sports organizations by providing funding, resources, and infrastructure to enable citizens to participate in organized sports activities. Individuals can develop regular exercise habits by raising their awareness of exercise. The strengths of our study include the large sample size, with a representative population and the use of a validated measure of physical activity (51). Our study has some potential limitations. First, the surveys were not conducted annually, which could have allowed for a more detailed and quantitative analysis of the trend. Second, all sociodemographic characteristics and physical activity data were based on self-reported information, which is likely to have introduced bias. Third, the study had a cross-sectional design; hence the causality of relationships between sociodemographic factors and regular exercise could not be determined. However, this study has four different time points, and the time interval can reflect the trend of change.

## Conclusion

Our study showed a significant increase in the overall rate of regular exercise in adults in Jiangsu Province over the 8 year period from 2010 to 2018; however, the rate decreased among retired participants. Being older, living in urban areas, having a higher education level, engaging in non-agriculture-related work, having a higher income, having a higher BMI, having a chronic disease at baseline, former smoking, and ever drinking were all positively associated with regular exercise from 2010 to 2018. Interventions targeted at population subgroups with low regular exercise rates, or those showing decreasing trends in regular exercise, are warranted.

## Data availability statement

The raw data supporting the conclusions of this article will be made available by the authors, without undue reservation.

## Ethics statement

The protocol for the 2015 chronic disease and risk factor surveillance survey was approved by the ethics review committee of the Chinese Center for Disease Control and Prevention (CDC), and all other surveys were approved by the ethical committee of National Center for Chronic and Noncommunicable Disease Control and Prevention (NCNCD), China CDC. The patients/participants provided their written informed consent to participate in this study.

## Author contributions

JS and JY designed the research. JY performed the data analyses and drafted the manuscript. JS revised the data analysis. JS, YQ, RT, JY, and SL conducted field investigations and data collection. SL, JZ, and MW revised the manuscript critically for important intellectual content. All authors contributed to the article and approved the submitted version.

## Conflict of interest

The authors declare that the research was conducted in the absence of any commercial or financial relationships that could be construed as a potential conflict of interest.

## Publisher’s note

All claims expressed in this article are solely those of the authors and do not necessarily represent those of their affiliated organizations, or those of the publisher, the editors and the reviewers. Any product that may be evaluated in this article, or claim that may be made by its manufacturer, is not guaranteed or endorsed by the publisher.

## Publisher’s note

All claims expressed in this article are solely those of the authors and do not necessarily represent those of their affiliated organizations, or those of the publisher, the editors and the reviewers. Any product that may be evaluated in this article, or claim that may be made by its manufacturer, is not guaranteed or endorsed by the publisher.
