# Supplementary material for: Trends in the rate of regular exercise among adults: results from chronic disease and risk factor surveillance from 2010 to 2018 in Jiangsu, China
Source: Front Public Health. 2023 Jun 15;11:1089587. doi: 10.3389/fpubh.2023.1089587 (PMC10308382; doi:10.3389/fpubh.2023.1089587)
Supplement: Supplementary file 1 [file Table_1.DOCX]

Supplementary Material

| Supplementary Table 1 Trends in weighted rate of regular exercise, CDRFS 2010-2018^a^ | | | | | |
| --- | --- | --- | --- | --- | --- |
|  | Rate, % (95% CI) | | | |  |
| Characteristic | 2010 (n=8374) | 2013 (n=8302) | 2015 (n=8372) | 2018 (n=8400) | *P* for trend^b^ |
| Overall | 12.28 (9.11-15.45) | 16.02 (12.57-19.47) | 19.02 (15.49-22.54) | 21.47 (17.26-25.69) | 0.009 |
| Gender |  |  |  |  |  |
| Female | 11.07 (7.90-14.24) | 14.41 (10.66-18.16) | 17.88 (13.97-21.78) | 19.18 (15.55-22.81) | 0.013 |
| Male | 13.40 (9.71-17.08) | 17.67 (13.98-21.36) | 20.08 (16.69-23.48) | 23.75 (18.58-28.92) | 0.012 |
| Age, years |  |  |  |  |  |
| 18-<45 | 11.85 (8.33-15.37) | 14.28 (10.76-17.80) | 19.58 (15.68-23.48) | 24.00 (20.18-27.81) | 0.001 |
| 45-<60 | 12.45 (8.95-15.95) | 17.03 (13.65-20.41) | 19.24 (15.17-23.30) | 21.26 (16.50-26.02) | 0.027 |
| ≥60 | 13.33 (9.41-17.26) | 18.94 (13.96-23.92) | 17.77 (13.81-21.72) | 18.64 (13.79-23.49) | 0.342 |
| Residence |  |  |  |  |  |
| Rural | 7.95 (2.83-13.07) | 10.62 (5.96-15.27) | 11.51 (7.06-15.96) | 15.42 (10.64-20.20) | 0.017 |
| Urban | 15.33 (10.46-20.20) | 19.03 (14.22-23.84) | 24.41 (18.76-30.06) | 25.83 (19.92-31.73) | 0.034 |
| Education |  |  |  |  |  |
| No formal education | 3.99 (2.44-5.54) | 8.79 (5.02-12.56) | 7.46 (4.08-10.84) | 11.90 (6.91-16.89) | 0.003 |
| Primary | 8.65 (5.99-11.31) | 11.69 (7.98-15.40) | 11.71 (9.51-13.91) | 14.61 (11.55-17.66) | 0.009 |
| Secondary | 13.95 (11.06-16.84) | 17.63 (14.13-21.13) | 21.63 (18.74-24.52) | 23.31 (19.41-27.22) | 0.007 |
| College or higher | 24.62 (15.87-33.37) | 26.15 (22.16-30.14) | 28.77 (21.95-35.59) | 32.77 (26.96-38.58) | 0.064 |
| Occupation |  |  |  |  |  |
| Agriculture-related | 3.71 (2.18-5.23) | 8.55 (4.64-12.45) | 7.86 (6.10-9.63) | 14.85 (10.80-18.90) | <0.001 |
| Manual work | 9.32 (4.82-13.81) | 16.92 (11.58-22.26) | 18.26 (14.72-21.80) | 19.52 (11.84-27.21) | 0.018 |
| Non-manual work | 15.33 (10.92-19.73) | 18.65 (15.56-21.74) | 20.74 (16.58-24.91) | 22.98 (18.99-26.96) | 0.027 |
| Not working | 13.03 (6.75-19.31) | 10.07 (4.10-16.04) | 11.41 (7.77-15.06) | 14.84 (11.77-17.91) | 0.404 |
| Retired | 33.79 (28.61-38.97) | 38.67 (32.15-45.19) | 35.77 (30.40-41.14) | 29.78 (24.21-35.36) | 0.071 |
| Annual household income, ¥ |  |  |  |  |  |
| <30,000 | 8.80 (5.82-11.79) | 11.57 (7.22-15.92) | 11.95 (8.56-15.34) | 17.50 (13.82-21.18) | 0.002 |
| 30,000-<60,000 | 13.68 (9.92-17.44) | 15.92 (11.57-20.27) | 16.92 (14.24-19.60) | 18.19 (14.28-22.11) | 0.093 |
| ≥60,000 | 18.49 (14.43-22.54) | 18.19 (14.83-21.55) | 23.76 (18.39-29.14) | 24.56 (18.55-30.57) | 0.065 |
| Refuse to answer/Unknown | 11.55 (6.68-16.42) | 15.61 (11.52-19.70) | 15.88 (11.37-20.39) | 19.17 (15.21-23.14) | 0.092 |
| BMI categories^c^ |  |  |  |  |  |
| Underweight | 8.65 (3.47-13.83) | 10.78 (4.86-16.71) | 14.03 (6.68-21.37) | 13.74 (3.97-23.52) | 0.293 |
| Normal | 11.41 (8.12-14.70) | 14.98 (11.99-17.98) | 18.14 (13.82-22.45) | 21.02 (16.37-25.67) | 0.009 |
| Overweight | 13.55 (10.15-16.94) | 17.40 (13.22-21.57) | 20.69 (16.66-24.71) | 22.12 (17.64-26.60) | 0.020 |
| Obesity | 12.49 (7.88-17.11) | 16.49 (10.36-22.61) | 17.76 (15.39-20.13) | 21.85 (16.82-26.88) | 0.015 |
| Self-reported chronic disease |  |  |  |  |  |
| No | 10.96 (7.83-14.09) | 14.12 (10.83-17.41) | 17.67 (13.59-21.75) | 20.83 (16.92-24.74) | 0.005 |
| Yes | 16.96 (13.11-20.80) | 21.87 (17.99-25.75) | 22.01 (19.02-24.99) | 22.53 (17.37-27.69) | 0.191 |
| Smoking |  |  |  |  |  |
| Never | 11.94 (8.65-15.23) | 15.40 (11.77-19.04) | 18.97 (14.63-23.30) | 20.79 (16.53-25.05) | 0.014 |
| Former | 17.56 (13.50-21.63) | 22.53 (18.16-26.89) | 23.42 (19.05-27.79) | 25.11 (17.63-32.59) | 0.074 |
| Current | 12.07 (8.37-15.78) | 16.52 (12.97-20.07) | 18.10 (15.60-20.60) | 22.32 (18.33-26.31) | 0.006 |
| Drinking |  |  |  |  |  |
| Never | 11.18 (8.25-14.10) | 15.08 (11.26-18.90) | 17.26 (13.09-21.44) | 20.05 (15.63-24.47) | 0.015 |
| Ever, 30 days ago | 14.33 (10.12-18.54) | 16.95 (12.23-21.67) | 20.95 (17.40-24.51) | 23.29 (18.43-28.15) | 0.016 |
| Ever, within 30 days | 13.00 (7.81-18.20) | 20.65 (15.27-26.04) | 22.02 (17.59-26.45) | 23.49 (18.58-28.40) | 0.034 |
| Region |  |  |  |  |  |
| South | 16.92 (14.74-19.10) | 22.33 (17.79-26.87) | 26.01 (18.78-33.24) | 26.07 (17.90-34.23) | 0.029 |
| Central | 4.79 (0.18-9.40) | 10.27 (3.40-17.14) | 15.34 (7.26-23.43) | 16.52 (9.53-23.50) | 0.039 |
| North | 11.03 (2.14-19.93) | 12.26 (3.01-21.50) | 12.45 (7.09-17.81) | 18.57 (11.91-25.23) | 0.082 |
| Abbreviations: BMI, body mass index; CDRFS, Chronic Disease and Risk Factor Surveillance. ^a^ Weighted estimates and 95%CIs were estimated for each survey. ^b^ P for trend was calculated using the year of each survey as a continuous variable. ^c^ Underweight (<18.5 kg/m^2^), normal (18.5 kg/m^2^-23.9 kg/m^2^), overweight (24.0 kg/m^2^-27.9 kg/m^2^), and obesity (≥28.0 kg/m^2^). | | | | | |

| Supplementary Table 2 Association between regular exercise (yes vs. no) and sociodemographic factors using separate data of CDRFS 2010-2018^a^ (OR and 95% CI) | | | | |
| --- | --- | --- | --- | --- |
|  | 2010 | 2013 | 2015 | 2018 |
| Characteristic | OR (95% CI) | OR (95% CI) | OR (95% CI) | OR (95% CI) |
| Gender |  |  |  |  |
| Female | ref | ref | ref | ref |
| Male | 0.96 (0.78-1.17) | 0.92 (0.77-1.10) | 0.93 (0.79-1.10) | 1.09 (0.92-1.28) |
| Age, years |  |  |  |  |
| 18-<45 | ref | ref | ref | ref |
| 45-<60 | 1.27 (1.06-1.53) | 1.33 (1.13-1.57) | 1.16 (0.98-1.37) | 1.09 (0.92-1.30) |
| ≥60 | 1.27 (1.00-1.62) | 1.52 (1.24-1.87) | 1.01 (0.82-1.24) | 0.97 (0.79-1.19) |
| *P* for trend^b^ | 0.036 | <0.001 | 0.975 | 0.560 |
| Residence |  |  |  |  |
| Rural | ref | ref | ref | ref |
| Urban | 1.18 (0.99-1.40) | 1.37 (1.17-1.60) | 1.47 (1.26-1.72) | 1.68 (1.45-1.95) |
| Education |  |  |  |  |
| No formal education | ref | ref | ref | ref |
| Primary | 2.04 (1.54-2.73) | 1.27 (1.02-1.59) | 1.25 (0.99-1.59) | 1.05 (0.85-1.28) |
| Secondary | 2.82 (2.13-3.78) | 1.85 (1.49-2.31) | 2.16 (1.72-2.73) | 1.64 (1.34-2.01) |
| College or higher | 4.62 (3.24-6.61) | 2.96 (2.20-3.98) | 2.89 (2.18-3.85) | 2.71 (2.05-3.57) |
| *P* for trend^c^ | <0.001 | <0.001 | <0.001 | <0.001 |
| Occupation |  |  |  |  |
| Agriculture-related | ref | ref | ref | ref |
| Manual work | 2.00 (1.45-2.76) | 1.83 (1.46-2.30) | 1.53 (1.15-2.02) | 0.96 (0.73-1.27) |
| Non-manual work | 2.90 (2.31-3.68) | 1.67 (1.40-1.98) | 1.60 (1.31-1.97) | 1.11 (0.93-1.33) |
| Not working | 2.56 (1.58-4.02) | 0.98 (0.64-1.44) | 1.15 (0.83-1.58) | 0.82 (0.63-1.07) |
| Retired | 6.68 (5.07-8.84) | 3.29 (2.62-4.12) | 2.93 (2.31-3.73) | 1.62 (1.30-2.02) |
| Annual household income, ¥ |  |  |  |  |
| <30,000 | ref | ref | ref | ref |
| 30,000-<60,000 | 1.34 (1.13-1.59) | 1.20 (0.99-1.47) | 1.20 (0.97-1.47) | 0.90 (0.74-1.09) |
| ≥60,000 | 1.45 (1.17-1.78) | 1.16 (0.95-1.41) | 1.29 (1.07-1.57) | 1.03 (0.87-1.23) |
| Refuse to answer/Unknown | 1.24 (0.95-1.60) | 0.96 (0.77-1.21) | 1.13 (0.89-1.42) | 0.87 (0.70-1.07) |
| *P* for trend^d^ | 0.004 | 0.523 | 0.134 | 0.638 |
| BMI categories^e^ |  |  |  |  |
| Underweight | 0.74 (0.44-1.19) | 0.63 (0.38-1.00) | 0.67 (0.40-1.08) | 0.66 (0.41-1.04) |
| Normal | ref | ref | ref | ref |
| Overweight | 1.19 (1.02-1.39) | 1.16 (1.02-1.33) | 1.11 (0.98-1.27) | 1.06 (0.93-1.20) |
| Obesity | 1.11 (0.89-1.38) | 1.13 (0.94-1.35) | 0.96 (0.80-1.14) | 1.04 (0.88-1.21) |
| *P* for trend^f^ | 0.060 | 0.038 | 0.602 | 0.436 |
| Self-reported chronic disease |  |  |  |  |
| No | ref | ref | ref | ref |
| Yes | 1.37 (1.17-1.61) | 1.25 (1.09-1.44) | 1.18 (1.03-1.34) | 1.16 (1.03-1.31) |
| Smoking |  |  |  |  |
| Never | ref | ref | ref | ref |
| Former | 1.08 (0.80-1.46) | 1.29 (0.96-1.71) | 1.21 (0.95-1.54) | 1.03 (0.82-1.31) |
| Current | 0.86 (0.69-1.07) | 0.93 (0.77-1.13) | 0.81 (0.67-0.97) | 0.91 (0.77-1.09) |
| Drinking |  |  |  |  |
| Never | ref | ref | ref | ref |
| Ever, 30 days ago | 1.22 (1.02-1.46) | 1.16 (0.98-1.37) | 1.30 (1.12-1.51) | 1.11 (0.96-1.27) |
| Ever, within 30 days | 1.09 (0.83-1.42) | 1.40 (1.10-1.77) | 1.26 (1.04-1.53) | 0.97 (0.80-1.18) |
| Region |  |  |  |  |
| South | ref | ref | ref | ref |
| Central | 0.41 (0.32-0.53) | 0.48 (0.40-0.57) | 0.72 (0.61-0.85) | 0.78 (0.66-0.92) |
| North | 1.00 (0.84-1.18) | 0.73 (0.63-0.85) | 0.79 (0.67-0.93) | 1.29 (1.10-1.52) |
| Abbreviations: BMI, body mass index; CDRFS, Chronic Disease and Risk Factor Surveillance.  ^a^ Unweighted estimates and 95%CIs were estimated for each survey.  ^b^ *P* for trend over age was calculated using the median value of each category as a continuous variable.  ^c^ *P* for trend over education was calculated using education level as a continuous variable.  ^d^ *P* for trend over annual household income was calculated using the median value of each category as a continuous variable.  ^e^ Underweight (<18.5 kg/m^2^), normal (18.5 kg/m^2^-23.9 kg/m^2^), overweight (24.0 kg/m^2^-27.9 kg/m^2^), and obesity (≥28.0 kg/m^2^).  ^f^ *P* for trend over BMI categories was calculated using the median value of each category as a continuous variable. | | | | |


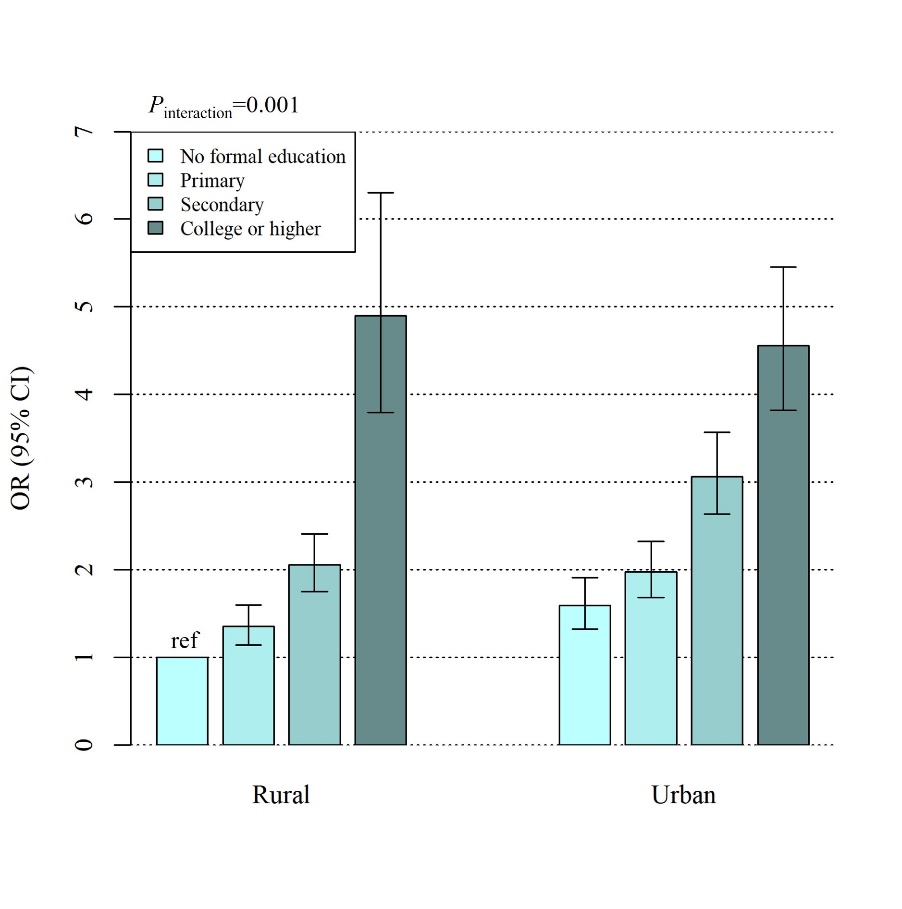


Supplementary Figure 1 The interaction between residence and education in multivariable regression


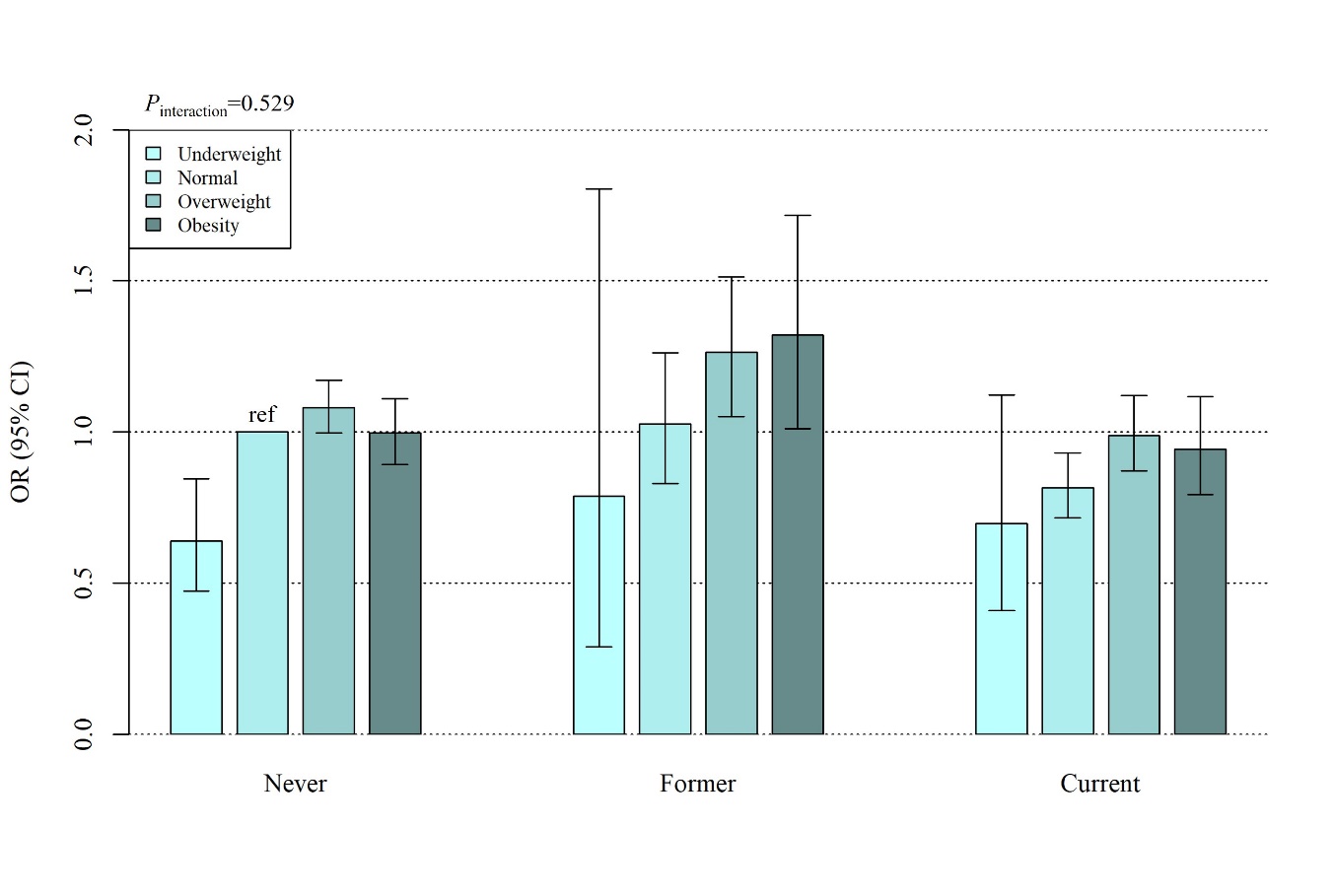


Supplementary Figure 2 The interaction between smoking and BMI in multivariable regression
